# Effects of Trichostatin A on drug uptake transporters in primary rat hepatocyte cultures

**DOI:** 10.17179/excli2015-220

**Published:** 2015-05-05

**Authors:** Eva Ramboer, Vera Rogiers, Tamara Vanhaecke, Mathieu Vinken

**Affiliations:** 1 In Vitro Toxicology and Dermato-cosmetology research group, Center for Pharmaceutical Research, Faculty of Medicine and Pharmacy, Vrije Universiteit Brussel

**Keywords:** epigenetics, Trichostatin A, drug transporters, primary hepatocyte culture

## Abstract

The present study was set up to investigate the effects of Trichostatin A (TSA), a prototypical epigenetic modifier, on the expression and activity of hepatic drug uptake transporters in primary cultured rat hepatocytes. To this end, the expression of the sinusoidal transporters sodium-dependent taurocholate cotransporting polypeptide (Ntcp) and organic anion transporting polypeptide 4 (Oatp4) was monitored by real-time quantitative reverse transcriptase polymerase chain reaction analysis and immunoblotting. The activity of the uptake transporters was analyzed using radiolabeled substrates and chemical inhibitors. Downregulation of the expression and activity of Oatp4 and Ntcp was observed as a function of the cultivation time and could not be counteracted by TSA. In conclusion, the epigenetic modifier TSA does not seem to exert a positive effect on the expression and activity of the investigated uptake transporters in primary rat hepatocyte cultures.

## Abbreviations

ABC, ATP binding cassette; B2m, beta-2 microglobulin; BSA, bovine serum albumin; DNA, complementary deoxyribonucleic acid; DNMT, DNA methyltransferase; DMSO, dimethylsulfoxide; Gapdh, glyceraldehyde-3-phosphate dehydrogenase; HDAC, histone deacetylase; Hnf-1a, hepatocyte nuclear factor-1a; Hmbs, hydroxymethylbilane synthase; (m)RNA, (messenger)ribonucleic acid; Ntcp, sodium-dependent taurocholate cotransporting polypeptide; Oatp4, organic anion transporting polypeptide 4; PBS, phosphate buffered saline; RT-qPCR, real-time quantitative reverse transcriptase polymerase chain reaction; SLC, solute carrier; TBS-T, Tris-buffered saline solution; TSA, Trichostatin A; Ubc, ubiquitin C.

## Introduction

Over the years, numerous animal studies have been carried out to determine the pharmaco-toxicological properties of new chemical entities. However, their use has been strongly criticized and with the introduction of the 3Rs concept of Russell and Burch in 1959[31], more attention has been paid to the establishment of alternative *in vitro *models (EMA, 2011[4], 1997[5]; Flecknell, 2002[6]; May et al., 2009[24]). Among the variety of liver-based *in vitro* models available today, cultures of primary hepatocytes are generally considered as the gold standard. Indeed, in these cells, the entire spectrum of biotransformation enzymes and drug transporters involved in hepatic drug clearance is expressed at an *in vivo*-like level, rendering this *in vitro* setting an excellent tool to evaluate drug metabolism and to predict drug-induced liver toxicity (Hewitt et al., 2007[10]; Ramboer et al., 2013[28]; Ulvestad et al., 2011[35]; Vinken et al., 2012[37]). However, primary hepatocyte cultures cope with dedifferentiation, which also negatively affects drug transporter expression (Jigorel et al., 2005[15]; Luttringer et al., 2002[23]; Rippin et al., 2001[29]). Accordingly, considerable focus has been put on the development of strategies to counteract this deteriorative process, in particular by mimicking the *in vivo *hepatic micro-environment (Hewitt et al., 2007[10]; Papeleu et al., 2002[26]). Cultivating rat hepatocytes between 2 layers of gelled collagen (*i.e.* sandwich configuration) has been reported to lead to a better retention of drug transporter expression (Liu et al., 1998[21]) and support the reestablishment of the canalicular network and proper localization of canalicular transporters, making this *in vitro *model often the reference system to study hepatobiliary transport (Ramboer et al., 2013[28]). However, in this *in vitro *model the expression and functionality of the drug uptake transporters are usually still considerably decreased in function of culture time (Jacobsen et al., 2011[14]; Jørgensen et al., 2007[16]; Kotani et al., 2011[19]; Tchaparian et al., 2011[33]). Since these conventional strategies do not tackle the actual causes of dedifferentiation, new approaches that affect dedifferentiation at the most upstream level of its regulation could thereby more effectively impact this process. In this respect, epigenetic modification of hepatocellular gene expression by histone deacetylase (HDAC) inhibitors, with Trichostatin A (TSA) as a prototype, can strongly favour the differentiated phenotype in primary rat hepatocyte cultures, including boosting of functional expression of phase I biotransformation enzymes (Henkens et al., 2007[9]). Yet, no information regarding the effect of HDAC inhibition on drug transporter expression and functionality in primary hepatocyte cultures is available. Indeed, reports on the epigenetic regulation of drug transporter proteins are limited and mainly restricted to cancer research (Klaassen et al., 2011[18]). Nevertheless, it has been repeatedly demonstrated that in mice, epigenetic mechanisms, namely DNA methylation and/ or histone acetylation, are involved in liver-specific expression of drug transporters (Douet et al., 2007[3]; Imai et al., 2009[12], 2013[11]). The latter are specialized transmembrane proteins that belong either to the solute carrier (SLC) superfamily or to the ATP binding cassette (ABC) superfamily (Li et al., 2009[20]; Russel, 2010[30]; Shugarts and Benet, 2009[32]; Thompson, 2011[34]; Zhang et al., 2006[38]). SLC superfamily members, such as the organic anion transporting polypeptide 4 (Oatp4) and the sodium taurocholate cotransporting polypeptide (Ntcp), as well as their ABC counterparts, are frequently involved in drug-drug interactions and drug-induced toxicities (Giacomini et al., 2010[8]; Kato et al., 2002[17]; Thompson, 2011[34]; Zhang et al., 2006[38]). Hence, drug transporter research is nowadays routinely implemented in safety and efficacy assessment during the drug development process. This research area, at least in part, typically relies on *in vitro* experimentation (Ramboer et al., 2013[28]). The current study was set up to investigate whether epigenetic modification also affects the expression and functionality of drug uptake transporters *in vitro* and the TSA-optimized primary hepatocyte cultures could represent a user-friendly alternative for the labour-intensive “sandwich” cultures for transporter research. 

## Materials and Methods

### Chemicals and reagents

TSA was supplied by Errant Gene Therapeutics (United States of America). The radiolabeled compounds ([3H] taurocholic acid (specific activity 5 Ci/mmol), [3H] estrone-3-sulfate (specific activity 45.6 Ci/mmol), scintillation vials and scintillation fluid (Ultima Gold MV) were purchased from Perkin Elmer (Belgium). All other chemicals were commercially available products of analytical grade and were obtained from Sigma (Belgium), unless specified otherwise. Probenecid and TSA were dissolved in dimethylsulfoxide (DMSO), with final DMSO concentrations not exceeding 1 % v/v.

### Hepatocyte isolation and cultivation

Procedures for the housing of rats as well as for the isolation and cultivation of hepatocytes were approved by the local Ethical Committee of the Vrije Universiteit Brussel (Belgium). Male outbred Sprague-Dawley rats, weighing 250-275 g (Charles River Laboratories, France) were kept under controlled environmental conditions with free access to food and water. Hepatocytes were isolated by use of a 2-step collagenase method (Papeleu et al., 2006[26]). Viable (≥ 85 %) hepatocytes were seeded on a plastic surface at a density of 0.57 x 10^5^ cells/cm^2^ (6-well plates - 9.6 cm^2^) or 0.82 x 10^5^ cells/cm^2^ (6 cm dish - 19.5 cm^2^) in William's medium E (Invitrogen, Belgium) supplemented with 7 ng/ml glucagon, 292 mg/ml L-glutamine, antibiotics (7.33 I.E./ml sodium benzyl penicillin, 50 µg/ml kanamycin monosulphate, 10 µg/ml sodium ampicillin, 50 µg/ml streptomycin sulphate) and 10 % v/v fetal bovine serum (Gibco, Belgium). Cell culture plates were placed in an incubator (37 °C, 5 % CO_2_) and after 4 hours, cell culture media were removed and replaced by serum-free medium supplemented with 25 µg/ml hydrocortisone sodium hemisuccinate and 0.5 µg/ ml insulin. All hepatocyte cultures were maintained in an incubator (37 °C, 5 % CO_2_) and cell culture media were replaced daily. The cultivation conditions used were as follows: monolayer cultures either exposed to 

*(i)* 25 µM TSA or 

*(ii)* 0.083 % v/v DMSO (solvent control), or 

*(iii)* untreated monolayer cultures. 

Samples were taken 4 hours after plating (T4) and on day 4 (D4) of the cultivation period.

### Real-time quantitative reverse transcriptase polymerase chain reaction (RT-qPCR) analysis

Cells were harvested by scraping, washed with ice-cold phosphate buffered saline (PBS) and pelleted. Total cellular ribonucleic acid (RNA) extraction, complementary deoxyribonucleic acid (cDNA) production and cDNA purification were carried out as outlined elsewhere (De Kock et al., 2012[2]). The RT-qPCR reaction mix and RT-qPCR conditions, using the StepOnePlus system (Applied Biosystems, Belgium), were established according to the manufacturer's instructions (Applied Biosystems, Belgium). Gene mixes were purchased form Applied Biosystems (Table 1(Tab. 1)). Selection of reliable housekeeping genes for normalization of the RT-qPCR data was done using qbasePLUS software (Biogazelle, Belgium), whereby beta-2 microglobulin (B2m), ubiquitin C (Ubc), hydroxymethylbilane synthase (Hmbs) and glyceraldehyde-3-phosphate dehydrogenase (Gapdh) were selected as the most stable housekeeping genes (results not shown). The results were processed according to the 2^-ΔΔCT^ method (Livak and Schmittgen, 2001[22]) and relative messenger RNA (mRNA) expression levels of each drug transporter were expressed as fold changes normalized against the geometric means of all 4 housekeeping gene mRNAs and scaled against the mRNA expression level of T4 untreated monolayer cultures, arbitrarily set at 100 %. 

### Western blot analysis

Cells were harvested by scraping, washed with ice-cold PBS and pelleted. Total cellular protein extraction was performed as described elsewhere (Vinken et al., 2011[36]) and quantification was carried out according to the Bradford procedure using bovine serum albumin (BSA) as a standard (Bradford, 1976[1]). Proteins (50 μg) were heated, fractionated on sodium dodecyl sulphate polyacrylamide (7.5 % or 10 % w/v) and blotted afterwards onto nitrocellulose membranes (Amersham, United Kingdom) (Ntcp) or polyvinylidene difluoride membranes (Bio-Rad, Germany) (Oatp4). For Ntcp the subsequent steps were performed using the Supersignal Western Blot Enhancer kit (Pierce, Belgium). Membranes were blocked with 5 % w/v non-fatty milk in Tris-buffered saline solution (20 mM tris(hydroxymethyl) amino, 135 mM NaCl) containing 0.1 % v/v Tween 20 (TBS-T). Membranes were incubated overnight at 4 °C with primary antibodies directed against a specific drug transporter (Table 2(Tab. 2)), followed by incubation for 1 hour at room temperature with a horseradish peroxidase-conjugated secondary antibody (Dako, Denmark). Excess antibody was removed by washing the membranes several times with TBS-T. Detection of the proteins was carried out by means of an enhanced chemiluminescence Western blotting system (Pierce, Belgium). For semi-quantification of the results, blots were further incubated with a primary antibody against hepatocyte nuclear factor-1α (Hnf-1α) (Table 2(Tab. 2)), which was previously identified as a robust housekeeping protein in primary rat hepatocyte cultures (Henkens et al., 2007[9]). Blots were scanned and densitometric analyses were performed by using the Quantity One software (Bio-Rad, Germany). Transporter signals were normalized against the corresponding Hnf-1α signals and were expressed as percentage of the normalized transporter signals in T4 untreated monolayer cultures, arbitrarily set at 100 %.

### Sinusoidal uptake assay

Cell culture medium was removed and cells were washed twice and incubated for 10 minutes with transporter assay buffer at 37 °C [5.3 mM KCl, 1.1 mM KH_2_PO_4_, 0.8 mM MgSO_4_, 1.8 mM CaCl_2_, 11 mM D-glucose, 10 mM HEPES, and 136 mM N-methyl glucamine (sodium-free buffer) or 136 mM NaCl (sodium-containing buffer)] (Jigorel et al., 2005[15]). To study the activity of Ntcp and Oatp4, cells were incubated with transporter uptake buffer at 37 °C supplemented with the radiolabeled substrates (1 µCi/ml) [3H] taurocholic acid (0.2 µM) (Ntcp) and [3H] estrone-3-sulfate (0.02 µM) (Oatp4) either in the presence or absence of their inhibitors, namely sodium ions (Ntcp) and probenecid (1 mM) (Oatp4), respectively. Uptake was stopped by addition of ice-cold PBS containing 0.2 % w/v BSA (Poirier et al., 2008[27]). After a final washing step with ice-cold PBS, cells were lyzed with mammalian protein extraction reagent (Pierce, Belgium) and the intracellular accumulation of the radiolabeled substrate was measured using liquid scintillation counting (Tri-Carb, Perkin Elmer, Belgium). The total protein content from each well was determined by means of the Bradford method (Bradford, 1976[1]) with BSA as a standard and was used to normalize uptake activity. Passive transport was determined by performing experiments at 4 °C using cold substrate solutions (Poirier et al., 2008[27]). The difference in intracellular radioactive accumulation in the absence and presence of the respective inhibitors was considered to represent the transporter activity (Jigorel et al., 2005[15]). 

### Statistical analysis

Data are expressed as mean ± standard deviation of at least 3 independent experiments. Results were evaluated using a one-tailed or 2-tailed paired Student's *t*-test with level of significance p ≤ 0.05. In order to determine changes in function of the cultivation time, the expression and activity of transporters in T4 and D4 untreated monolayer cultures were analyzed. The effect related to the addition of DMSO or TSA to the culture medium was studied at each timepoint by comparing the results obtained in solvens control with untreated monolayers or TSA-treated hepatocytes with solvens control, respectively. 

## Results

### The effect of TSA on the mRNA expression of drug uptake transporters in primary rat hepatocyte cultures

**Figure 1:**
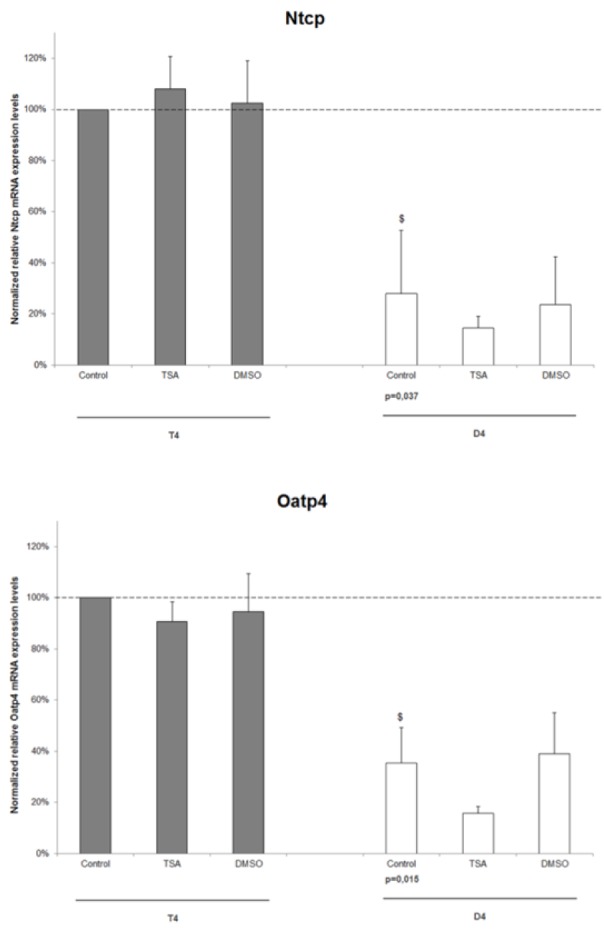
The effect of TSA on the mRNA expression level of drug uptake transporters in primary rat hepatocyte cultures. Freshly isolated rat hepatocytes were cultivated in 6 cm diameter dishes (19.5 cm^2^) as described in chapter "Hepatocyte isolation and cultivation”. Hepatocyte samples were taken 4 hours after cell plating (T4) and at day 4 of the cultivation period (D4). Samples were subjected to RT-qPCR analysis, using B2m, Gapdh, Hmbs, Ubc as a combination of housekeeping genes for normalization. Data are expressed as mean ± standard deviation of 3 independent experiments and normalized to the mRNA level of T4 untreated hepatocytes (arbitrarily set at 100 %, indicated with a dotted line). Statistical analyses were performed using a 2-tailed paired Student's *t*-test with p value ≤ 0.05 considered to be significantly different. ^$^p<0.05 when D4 untreated hepatocytes are compared with T4 counterparts. Samples collected 4 hours after cell plating are indicated with a gray bar and 4-day old cultured hepatocytes with a white bar. (D4, day 4 cultures; DMSO, dimethylsulfoxide; Nctp, sodium taurocholate cotransporting polypeptide; Oatp4, organic anion transporting polypeptide 4; T4, 4 hours cultures; TSA, Trichostatin A)

Previous research showed that in primary rat hepatocyte cultures 25 µM TSA positively affects the maintenance of cytochrome P450-mediated biotransformation capacity and the longevity of the cells (Henkens et al., 2007[9]). Because of these results, the same *in vitro *system was used to investigate whether this higher functionality is also associated with effects on drug uptake transporters. As such freshly isolated rat hepatocytes were cultured in a conventional monolayer configuration either in the absence or presence of 25 µM TSA for 4 days. Since DMSO (*i.e.* the TSA solvent) is known to favour the maintenance of the differentiated phenotype of primary hepatocytes (Isom et al., 1985[13]), a number of cultures were separately treated with 0.083 % v/v DMSO, being the concentration used as TSA solvent. RT-qPCR analysis was performed on T4 and D4 hepatocyte samples using B2m, Gapdh, Hmbs and Ubc as a combination of housekeeping genes for normalization (Figure 1(Fig. 1)). The mRNA levels of the uptake transporters Ntcp (p=0.037) and Oatp4 (p=0.015) significantly dropped as a function of the cultivation time in untreated hepatocytes. Furthermore, no clear effect of TSA or DMSO could be seen at both timepoints for the investigated transporters. 

### The effect of TSA on the protein expression of drug uptake transporters in primary rat hepatocyte cultures

**Figure 2:**
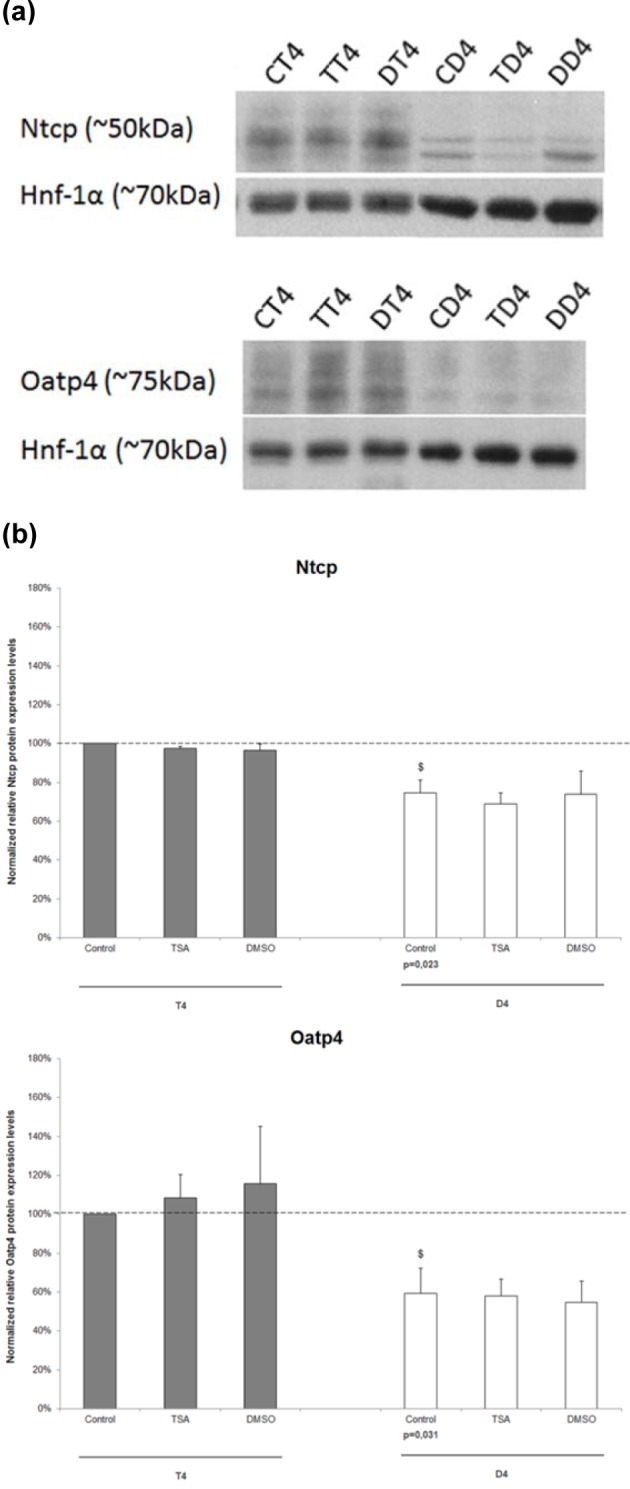
The effect of TSA on the protein expression levels of drug uptake transporters in primary rat hepatocyte cultures. Freshly isolated rat hepatocytes were cultivated in 6 cm diameter dishes (19.5 cm^2^) as described in chapter "Hepatocyte isolation and cultivation”. (a) Samples were taken 4 hours after cell plating (T4) and at day 4 of the cultivation period (D4) and subjected to Western blot analysis. Results of 3 independent experiments are shown. (b) Densitometric analysis of the expression of drug transporters. Transporter signals were normalized against the corresponding Hnf-1α signals and were expressed as a percentage of the normalized transporter signals in T4 untreated hepatocytes (arbitrarily set at 100 %, indicated with a dotted line). The results are shown as mean ± standard deviation of 3 independent experiments. Statistical analyses were performed using a 2-tailed paired Student's *t*-test with p values ≤ 0.05 considered to be significantly different. ^$^p<0.05 when D4 untreated hepatocytes are compared with T4 counterparts. Samples collected 4 hours after cell plating are indicated with a gray bar and 4-day old cultured hepatocytes with a white bar. (CD4, untreated hepatocytes D4; CT4, untreated hepatocytes T4; D4, day 4 cultures; DD4, DMSO-treated hepatocytes D4; DMSO, dimethylsulfoxide; DT4, DMSO-treated hepatocytes T4; Nctp, sodium taurocholate cotransporting polypeptide; Oatp4, organic anion transporting polypeptide 4; T4, 4 hours cultures; TD4, TSA-treated hepatocytes D4; TSA, Trichostatin A; TT4, TSA-treated hepatocytes T4)

In order to investigate whether the findings at the mRNA level are translated into similar protein modifications, semi-quantitative immunoblot analyses were performed on whole cell lysates, using Hnf-1α as a housekeeping protein (Figures 2a and b(Fig. 2)). The significant downregulation in Ntcp (p=0.023) and Oatp4 (p=0.031) expression as a function of the cultivation time, observed by RT-qPCR analysis, was confirmed by immunoblot analysis and no significant effects of TSA or DMSO could be seen at both timepoints for the investigated transporters.

### The effect of TSA on the activity of drug uptake transporters in primary rat hepatocyte cultures

To provide a complete picture of potential effects of TSA on uptake transporters in primary hepatocyte cultures, a number of activity assays were performed. In this respect, intracellular accumulation of radiolabelled substrates of Oatp4 ([3H] estrone-3-sulfate) and Ntcp ([3H] taurocholic acid) was assessed, both in the presence and absence of drug inhibitors, probenicid and sodium chloride, respectively. Results are presented as uptake of 1 µCi/ ml [3H] taurocholic acid and [3H] estrone-3-sulfate after 90 seconds incubation. The uptake of both substrates at that specific concentration was shown to be linear over this time range (supplementary Figures 1 and 2).

**Figure 3:**
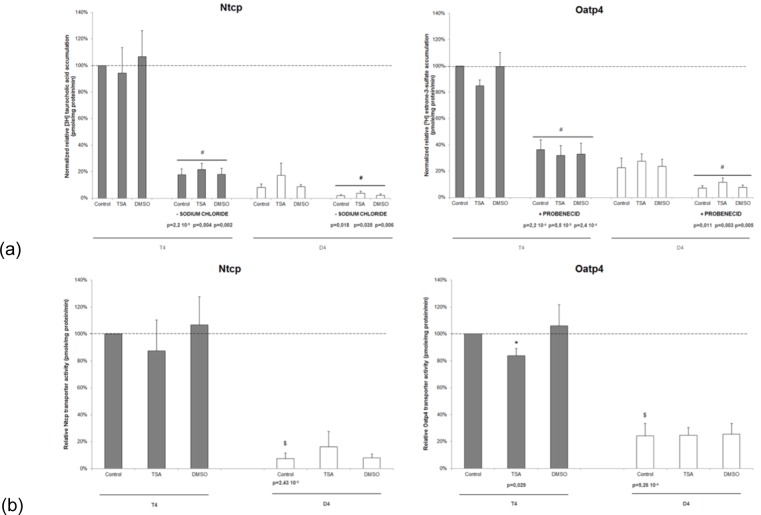
The effect of TSA on drug uptake transporter activity in primary rat hepatocyte cultures. Freshly isolated rat hepatocytes were cultivated in 6-well plates (9.6 cm^2^) as described in chapter "Hepatocyte isolation and cultivation”. Hepatocyte cultures were incubated for 90 seconds with 1 µCi/ml radioactive substrate [^3^H] taurocholic acid (0.2 µM) (Ntcp) and [^3^H] estrone-3-sulfate (0.02 µM) (Oatp4) either in the presence or absence of their inhibitors, namely sodium ions (136 mM) (Ntcp) and probenecid (1 mM) (Oatp4), respectively. (a) Representation of intracellular accumulation in different experimental settings. The results were expressed as mean ± standard deviation of 4 independent experiments and normalized for the intracellular accumulation measured in the untreated monolayer cultures in the absence of the respective inhibitor at T4 of cultivation (arbitrarily set at 100 %, indicated with a dotted line). Statistical analyses were performed using a one-tailed paired Student's t-test with p values ≤ 0.05 considered to be significantly different. ^#^p≤0.05, when compared to substrate accumulation in the absence of an inhibitor (b) Transporter activity, equivalent to substrate uptake in the absence of the respective inhibitor minus the uptake in the presence of the inhibitor, is determined at T4 and D4 of cultivation for Ntcp and Oatp4. These results were expressed as mean ± standard deviation of 4 independent experiments with the transporter activity of untreated monolayers 4 hours after cell plating arbitrarily set at 100 %. Statistical analyses were performed using a 2-tailed paired Student's t-test with p values ≤ 0.05 considered to be significantly different. ^$^p≤0.05, when D4 transporter activity is compared with functionality in T4 untreated monolayer cultures; *p≤0.05, when TSA treated hepatocytes are compared with solvens control cultures. Samples collected 4 hours after cell plating are indicated with a gray bar and 4-day old cultured hepatocytes with a white bar. ([3H], tritium; D4, day 4 cultures; DMSO, dimethylsulfoxide; Nctp, sodium taurocholate cotransporting polypeptide; Oatp4, organic anion transporting polypeptide 4; T4, 4 hours cultures; TSA, Trichostatin A)

The intracellular accumulation of the radioactive substrates underwent a clear downregulation in the presence of the respective inhibitors, indicating the proper functioning of Ntcp and Oatp4 in the 3 cultivation conditions at both timepoints (Figure 3a(Fig. 3)). However, the smaller inhibitory effect observed in D4 cultures compared to T4 cultures suggests reduced transporter functionality at this timepoint. Indeed, Ntcp and Oatp4 activity, represented by the difference in accumulation in the absence and presence of the corresponding inhibitor, was significantly decreased as a function of cultivation time. No effects related to the use of DMSO were seen at both timepoints. Only in T4 cultures, lower Oatp4 functionality (p=0.029) was observed in TSA-treated versus DMSO-treated hepatocytes (Figure 3b(Fig. 3)). 

## Discussion

The current study was set up to investigate whether TSA, a HDAC inhibitor earlier shown in our laboratory to maintain cytochrome P450-mediated biotransformation competence in primary cultured rat hepatocytes (Henkens et al., 2007[9]), also affects drug transporter-mediated phase 0 metabolism. Focus was hereby put on the sinusoidal uptake transporters Ntcp and Oatp4. When seeded in a conventional monolayer configuration, canalicular drug transporter mRNA levels in primary cultured rat hepatocytes are usually better maintained in comparison with their basolateral counterparts (Jigorel et al., 2005[15]; Luttringer et al., 2002[23]; Rippin et al., 2001[29]). In analogy with previous reports, downregulated mRNA expressions were observed for Ntcp and Oatp4 as a function of the cultivation time. Regarding TSA, no significant differences could be noticed in transporter mRNA quantities of TSA-treated and DMSO-treated monolayer cultures. Consequently, TSA does not seem to affect hepatic drug transporter gene expression, at least not in this type of *in vitro *system. 

Protein expression of drug transporters in conventional monolayer cultures has in general not been extensively investigated. In this study, the detected translational modulations comply with the observed variations in mRNA expression levels. As previously described (Liu et al., 1998[21]; Rippin et al., 2001[29]), Ntcp protein expression levels significantly decrease as a function of the cultivation time. A similar regulation was observed for Oatp4. To the best of our knowledge, no other reports on Oatp4 protein expression in conventional monolayer cultures have yet been published. In line with other studies (Jigorel et al., 2005[15]; Liu et al., 1998[21]; Rippin et al., 2001[29]), the activities of Ntcp and Oatp4 strongly declined with cultivation time. In general, DMSO had no effect on the expression and activity of the drug uptake transporters studied. Nevertheless, positive effects of DMSO on hepatocellular Ntcp and Oatp drug transporters activity in primary hepatocyte cultures have already been described by others (Jigorel et al., 2005[15]). It should be mentioned that in the current study, DMSO was used as the solvent for TSA and was kept as low as possible (0.083 % v/v). In the study of Isom and colleagues DMSO was present in much higher concentrations (2 % v/v) in order to reach an optimal maintenance of the hepatocyte differentiated status (Isom et al., 1985[13]). 

In conclusion, the epigenetic modifier TSA does not seem to exert an effect on the expression and activity of drug uptake transporters in primary rat hepatocyte cultures although it clearly affected the metabolizing capacity (Henkens et al., 2007[9]). Since it has been show that DNA methylation is involved in the liver-specific expression of the investigated drug transporters (Imai et al., 2009[12]; Imai et al., 2013[11]), it could be interesting to study the effect of DNA methyltransferase (DNMT) as alternative epigenetic modulatory mechanism to improve drug transporter expression and activity *in vitro*. Furthermore, using a combination of epigenetic modulators, namely HDACi and DNMT, could also be explored as it has already been demonstrated to have a synergetic positive effect on the albumin secretory capacity of primary rat hepatocyte cultures (Fraczek et al., 2012[7]).

## Notes

Tamara Vanhaecke and Mathieu Vinken are equally contributing as last authors.

## Acknowledgements

This work was financially supported by the grants of the Fund for Scientific Research-Flanders-Belgium (FWO-Vlaanderen) and the Vrije Universiteit Brussel (OZR-VUB). The authors are grateful to Ms. Tineke Vanhalewyn and Mr. Paul Claes for their dedicated technical assistance.

## Conflict of interest

The authors declare that they have no conflict of interest. 

## Supplementary Material

Supplementary figures

## Figures and Tables

**Table 1:**
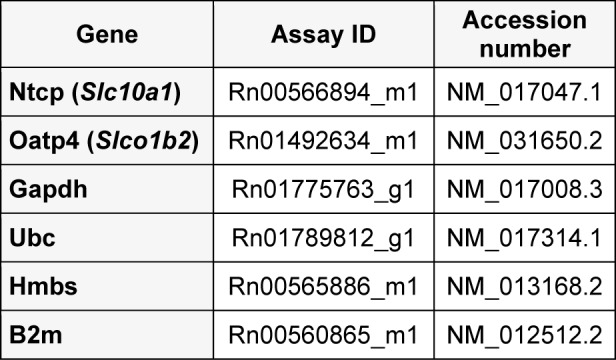
Gene expression assays used for qRT-PCR

**Table 2:**
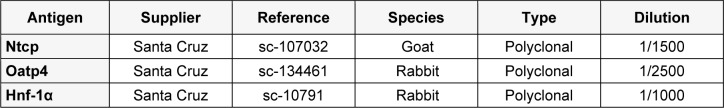
Primary antibodies used for Western blot analysis
