# Supplementary material for: Effects of Trichostatin A on drug uptake transporters in primary rat hepatocyte cultures
Source: EXCLI J. 2015 May 5;14:567–76. doi: 10.17179/excli2015-220 (PMC4669911; doi:10.17179/excli2015-220)
Supplement: Supplementary figures [file EXCLI-14-567-s-001.pdf]

EXCLI Journal 2015;14:567-576 – ISSN 1611-2156
Received: March 11, 2015, accepted: March 30, 2015, published: May 05, 2015

Supplementary material to:

EFFECTS OF TRICHOSTATIN A ON DRUG UPTAKE TRANSPORTERS IN PRIMARY RAT HEPATOCYTE CULTURES

Eva Ramboer*, Vera Rogiers, Tamara Vanhaecke°, Mathieu Vinken°

In Vitro Toxicology and Dermato-cosmetology research group, Center for Pharmaceutical Research, Faculty of Medicine and Pharmacy, Vrije Universiteit Brussel

° equally contributing last authors
***** Corresponding author: Eva Ramboer; In Vitro Toxicology and Dermato-cosmetology research group, Center for Pharmaceutical Research, Faculty of Medicine and Pharmacy, Vrije Universiteit Brussel, (VUB), Laarbeeklaan 103, B-1090 Brussels, Belgium.
E-mail: eramboer@vub.ac.be, +32.2.477.45.87

http://dx.doi.org/10.17179/excli2015-220

This is an Open Access article distributed under the terms of the Creative Commons Attribution License (http://creativecommons.org/licenses/by/4.0/).

EXCLI Journal 2015;14:567-576 – ISSN 1611-2156
Received: March 11, 2015, accepted: March 30, 2015, published: May 05, 2015

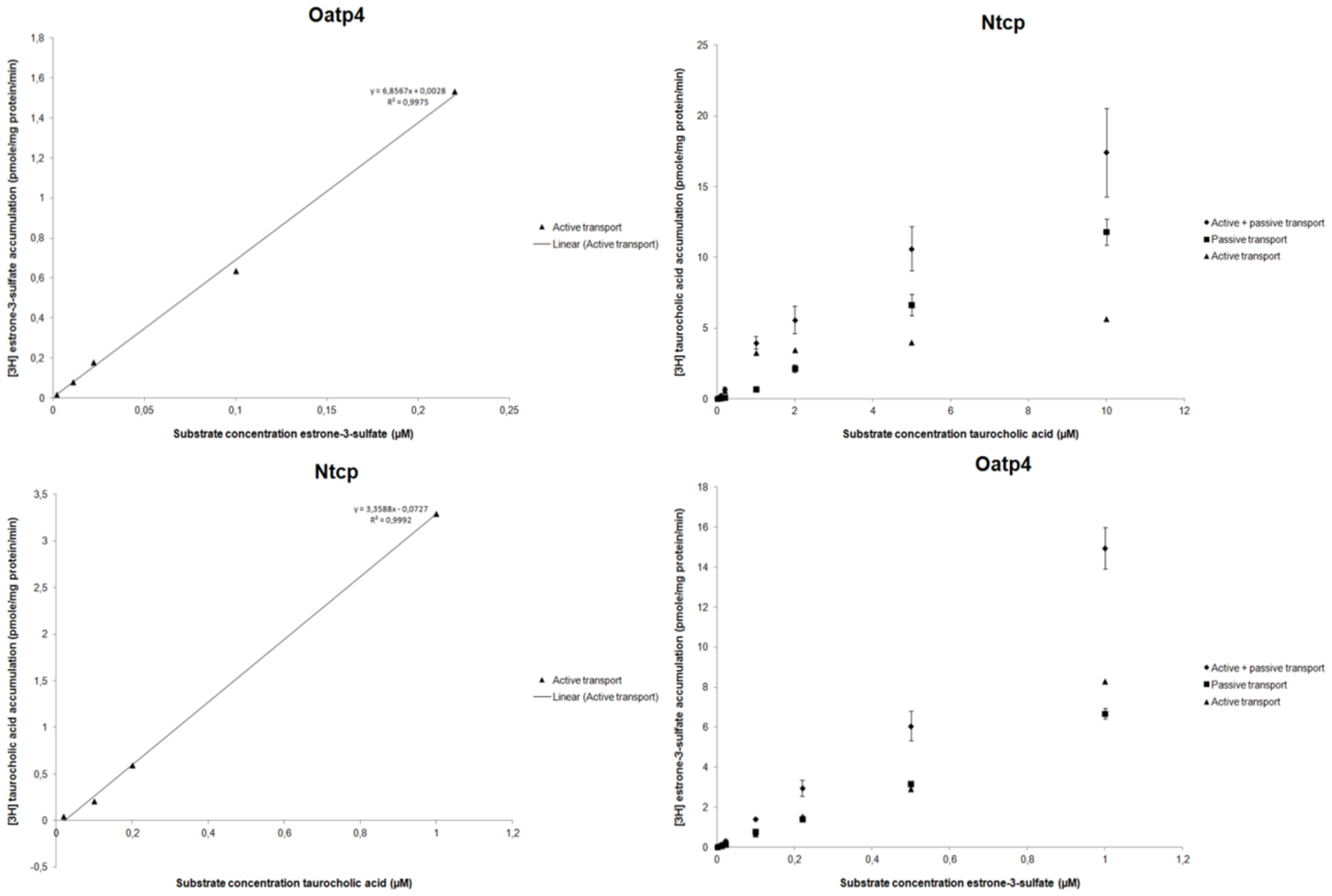

Supplementary Figure 1: Accumulation of radioactive substrates as a function of substrate concentration. Freshly isolated rat hepatocytes were cultivated in 6-well plates (9.6 cm^2) as described in “2.2 Hepatocyte isolation and cultivation”. Day 4 non-treated hepatocyte cultures were incubated for 90 seconds with a range of substrate concentrations, namely 0,02-10 µM [3H] Taurocholic acid (Ntcp) and 0,0022-1 µM [3H] Estrone-3-sulfate (Oatp4) either at 37 °C (active and passive transport) or 4 °C (passive transport). Active transport, equivalent to substrate uptake values at 37 °C minus uptake values at 4 °C was determined (n=1, 3 technical repeats). ([3H], tritium; D, day; Nctp, sodium taurocholate cotransporting polypeptide; Oatp4, organic anion transporting polypeptide 4)

EXCLI Journal 2015;14:567-576 – ISSN 1611-2156
Received: March 11, 2015, accepted: March 30, 2015, published: May 05, 2015

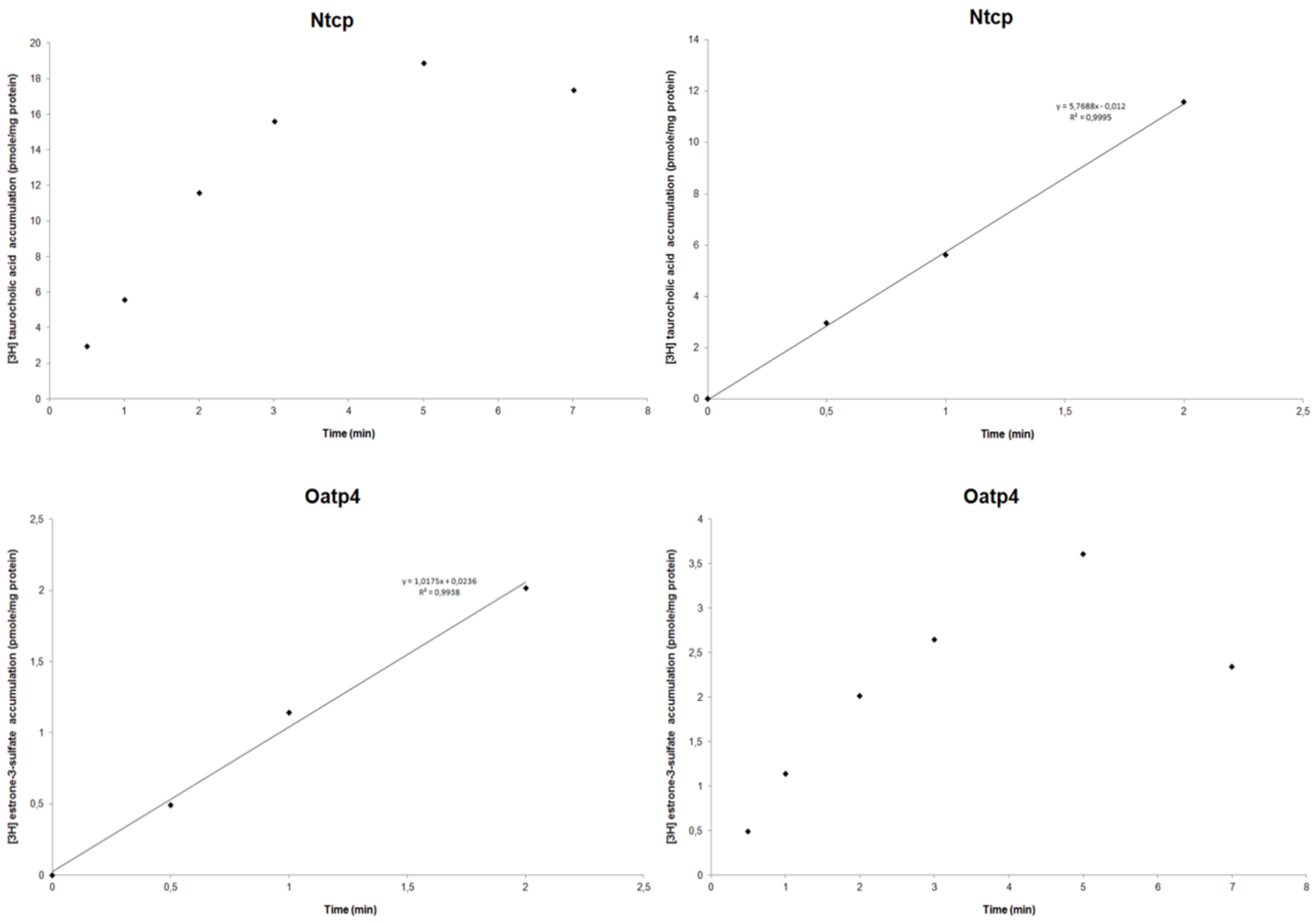

Supplementary Figure 2: Accumulation of radioactive substrates as a function of incubation time. Freshly isolated rat hepatocytes were cultivated in 6-well plates (9.6 cm^2) as described in “2.2 Hepatocyte isolation and cultivation”. Day 1 non-treated hepatocyte cultures were incubated for 0.5, 1, 2, 3, 5 and 7 minutes with 1 µCi/ml [3H] Taurocholic acid (0,2 µM) (Ntcp) or [3H] Estrone-3-sulfate (0,02 µM) (Oatp4) (n=1, 1 technical repeat) ($[^3H]$, tritium; D, day; Nctp, sodium taurocholate cotransporting polypeptide; Oatp4, organic anion transporting polypeptide 4)
